# Composite Bioinks With Mesoporous Bioactive Glasses—A Critical Evaluation of Results Obtained by *In Vitro* Experiments

**DOI:** 10.3389/fbioe.2021.767256

**Published:** 2022-01-11

**Authors:** Vera Guduric, Johannes Wieckhusen, Anne Bernhardt, Tilman Ahlfeld, Anja Lode, Chengtie Wu, Michael Gelinsky

**Affiliations:** ^1^ Centre for Translational Bone, Joint and Soft Tissue Research, University Hospital Carl Gustav Carus and Faculty of Medicine, Technische Universität, Dresden, Germany; ^2^ State Key Laboratory of High Performance Ceramics and Superfine Microstructure, Shanghai Institute of Ceramics, Chinese Academy of Sciences, Shanghai, China

**Keywords:** mesoporous bioactive glass, calcium, magnesium, composite bioinks, data interpretation, viability, osteogenic differentiation, ion release

## Abstract

Besides osteoconductivity and a high degradation rate, mesoporous bioactive glasses (MBGs) are specific for their highly ordered channel structure and high specific surface area, making them suitable as drug and/or growth factor delivery systems. On the other hand, the mesoporous channel structure and MBG composition can have an effect on common cell evaluation assays, leading to inconclusive results. This effect is especially important when MBG is mixed in composite bioinks, together with cells. Additionally, the hydrogel component of the ink can influence the degradation of MBG, leading to different ion releases, which can additionally affect the analyses. Hence, our aim here was to show how the MBG structure and composition influence common cell viability and differentiation assays when calcium (Ca)- or magnesium (Mg)-containing glass is part of an alginate-based composite bioink. We suggested pre-labeling of cells with DiI prior to bioprinting and staining with calcein-AM to allow identification of metabolically active cells expressing signals in both green and red channels, allowing the use of fluorescence imaging for cell viability evaluations in the presence of high amounts (7 wt %) of MBGs. The release and uptake of ions during degradation of CaMBG and MgMBG were significantly changed by alginate in the composite bioinks, as confirmed by higher release and uptake from bulk glasses. Additionally, we detected a burst release of Mg^2+^ from composites only after 24 h of incubation. Furthermore, we demonstrated that released ions and the mesoporous channel structure affect the measurement of lactate dehydrogenase (LDH) and alkaline phosphatase activity (ALP) in bioprinted composite scaffolds. Measured LDH activity was significantly decreased in the presence of CaMBG. On the other hand, the presence of MgMBG induced increased signal measured for the ALP. Taken together, our findings show how composite bioinks containing MBGs can interfere with common analyses, obtaining misleading results.

## 1 Introduction

Bioactive glasses (BGs) are widely used for dental and orthopedic applications, thanks to their high degradation rate and capability to induce the formation of nanocrystalline calcium phosphate structures as the main inorganic component of bone matrix (Baino et al., 2018). As a special type of BG, mesoporous bioactive glasses (MBGs) exhibit a highly ordered mesoporous channel structure and high specific surface area, making them suitable for loading of drugs and/or growth factors and acting as delivery systems (Wu and Chang, 2012). Furthermore, MBGs can be modified with different bioactive metal ions, assessing desired therapeutic effects after release (Zhu et al., 2021). A promising way to broaden MBG applicability is to use it in extrusion-based 3D (bio)printing that allows fabrication of volumetric bone tissue engineering constructs by processing pasty biomaterials (inks) or cell–biomaterial mixtures (bioinks). As particulate material, MBGs need to be combined with established (bio)inks, such as poly (vinyl alcohol) (Wu et al., 2011), calcium phosphate cements (Kauschke et al., 2018; Richter et al., 2019), alginate/methylcellulose (Guduric et al., 2021) and gelatin methacrylate (Tavares et al., 2021), to make them printable.

Water-rich biopolymers that constitute hydrogels are commonly used for bioinks. Hydrogels provide structural support for cells as a suitable biochemical and biophysical environment (Li et al., 2020). Additionally, hydrogels possess sufficient viscosity and shear-thinning behavior that are prerequisites for extrusion-based 3D bioprinting. Among a wide range of established hydrogels, the combination of ionically crosslinkable alginate (alg) and water-soluble, viscosity-enhancing methylcellulose (MC) was shown to be very useful for bioprinting (Ahlfeld et al., 2020b). An excellent print fidelity was achieved by the addition of 9 wt% MC to 3 wt% alg dissolved in phosphate-buffered saline (PBS) or human blood plasma, maintaining viability and functionality of human mesenchymal stem cells (hMSCs), rat pancreatic islets, chondrocytes, or human pre-osteoblasts (hOBs) (Schütz et al., 2017; Duin et al., 2019; Hodder et al., 2019; Ahlfeld et al., 2020a; Kilian et al., 2020). In composites of algMC with MBG, the addition of up to 10 wt% of MBG, in contrast to up to 1.5 wt% described in literature (Heras et al., 2020; Zhu et al., 2020; Tavares et al., 2021), resulted in an increased viscosity that has been counteracted by reducing the MC content to 6 wt%, maintaining shape fidelity and facilitating bioprinting of embedded hMSCs (Guduric et al., 2021). Furthermore, MBG can be modified with different therapeutic metal ions, for example Sr^2+^ and Zn^2+^, to stimulate cellular processes. Additionally, it is possible to control printing properties by adjusting MBG and MC content. Taken together, these properties make the system modular and adaptable to different indications.

Besides the mentioned advantages of MBG addition into bioinks, various difficulties are faced when performing experiments to evaluate certain properties of cells within the bioink. To the best of our knowledge, there are no data described in the literature about the evaluation of cell viability or morphology using fluorescence-based staining procedures in the presence of MBG. From our own experience, the main problem is that MBG particles express signals in respective channels after staining with specific fluorescent reagents, for example calcein-AM (staining of live cells) or ethidium homodimer-1 (staining of dead cells), making distinguishing of cells from glass particles very difficult or even impossible. High binding capacity of MBG, due to high specific surface area (mesoporous channel structure), which is favorable for drug and growth factor loading, is a disadvantage for analytics as dyes or cellular components released after the lysis are expected to bind. Moreover, when ion release from MBG or MBG-containing composites is investigated, the multicomponent bioink itself has a considerable impact on the outcome. These problems to obtain reliable results for the analysis of MBG-containing bioinks *in vitro* were not addressed in the literature by now. Therefore, our objective here is to show how MBG, embedded within an algMC bioink matrix, can affect assays of cell viability and differentiation. To this end, two different MBGs (modified with either Ca^2+^ or Mg^2+^) were synthesized and characterized, and printing properties of composites from algMC and MBG were adjusted. Bioprinted constructs with primary human pre-osteoblasts were generated and we paid attention on i) the observation of metabolically active cells in MBG–algMC composite bioinks by fluorescence microscopy, ii) the effect of the MBG structure and release products on investigation of lactate dehydrogenase (LDH) and alkaline phosphatase (ALP) activity after cell lysis and iii) the effect of algMC bioink on ion release/uptake by degrading of MBG. Magnesium was chosen for substitution of MBG because it is not a strong crosslinker of alginate and was, therefore, not expected to extremely affect rheological properties of the composite bioink. Furthermore, Mg^2+^ was shown to have osteostimulatory effects (Sheikh et al., 2015; Ramirez-Rodríguez et al., 2017; Gu et al., 2019).

## 2 Materials and Methods

### 2.1 MBG Synthesis and Characterization of the Mesoporous Structure

MBG powders containing 80, 5, and 15 mol% of silicon, phosphorus and calcium or magnesium, respectively, were synthesized in an evaporation-induced self-assembly process (EISA) using the protocol by Zhu et al. (2008). In brief, 4 g of pluronic (P123, *Mw* = 5,800, Sigma-Aldrich, Steinheim, Germany) was dissolved in 60 g of 96% ethanol and stirred for 1 h at room temperature. After that, 6.7 g of tetraethyl orthosilicate (99%, Sigma-Aldrich, Steinheim, Germany), 0.73 g of triethyl phosphate (99.8%, Sigma-Aldrich, St. Louis, MO, United States), 1.4 g of calcium nitrate tetrahydrate (for Ca-substituted MBG, CaMBG) or 1.52 g of magnesium nitrate hexahydrate (for Mg-substituted MBG, MgMBG) (both from Merck, Darmstadt, Germany) dissolved in 6 ml of deionized water, and 1 g of 0.5 M HCl were added, and the solution was stirred for 24 h. Then, the solution was distributed in petri dishes and dried at room temperature to allow evaporation of ethanol. The obtained gel was dried at 60°C for 3–4 h and ground and calcinated at 700°C, with a ramp of 2°C per minute. The obtained powder was ground to obtain particles smaller than 45 µm to allow final extrusion through a nozzle with an outlet diameter of 410 µm as needed for the 3D (bio)printing process. At the end, MBGs were sterilized by gamma irradiation (25 kGy). The mesoporous channel structure of both CaMBG and MgMBG was examined using the Tecnai TF30 G2 FED-TEM (ThermoFisher Scientific, formerly FEI, United States) transmission electron microscope (TEM) at 300 kV acceleration voltage with the standard single tilt holder. The images were captured on a Gatan Oneview (Gatan, CA, United States), and channel size was measured with ImageJ (1.44p, National Institutes of Health, Bethesda, MA, United States).

### 2.2 Effect of MBG Addition on Printing Properties of Composite Bioinks

#### 2.2.1 Preparation of Composite Bioinks

Composite (bio)inks were prepared by adding MBG in already established 3–6 algMC blend (Guduric et al., 2021). In brief, a 3 wt% alg solution was prepared by dissolving alginic acid sodium salt from brown algae (Sigma-Aldrich, Steinheim, Germany) in phosphate-buffered saline (PBS), stirred overnight, and autoclaved. Autoclaved methylcellulose (MC) powder (Sigma-Aldrich, Steinheim, Germany) was added into the alg solution to obtain a final concentration of 6 wt%. After mixing with a spatula, the blend was left for 30 min to allow swelling of MC. CaMBG or MgMBG were added into the algMC blend prior to printing to obtain a final concentration of 7 wt%, which was established as a suitable one for printing and biological properties of composite bioinks (Guduric et al., 2021).

#### 2.2.2 3D Printing of Composite Scaffolds and Investigation of Shape Fidelity

3D printing of scaffolds was performed using the BioScaffolder 3.1 (GeSiM mbH, Radeberg, Germany) by extruding inks (MBG-free and composites containing CaMBG or MgMBG) through conical nozzles with an outlet diameter of 410 µm. Four-layered square-shaped scaffolds (1 cm × 1 cm) were printed by material strand deposition, with a change of strand orientation of 90° between the layers, using parameters listed in the Table 1. After printing, each scaffold was placed in 100 mM CaCl_2_ solution for 10 min to crosslink alginate. After removing the crosslinking solution, the scaffolds were imaged with a stereomicroscope (Leica M205 equipped with DFC295 camera, Wetzlar, Germany). Strand widths were measured with ImageJ and compared to the theoretical value (410 µm).

**TABLE 1:** Parameters for 3D printing of MBG-free 3–6 algMC bioink and composites containing 7 wt% of CaMBG or MgMBG.

MBG	Pressure (kPa)	Speed (mm/s)
MBG-free	50	10
CaMBG	130	5
MgMBG	80	7

### 2.3 Ion Exchange of MBG and MBG Composites With Cell Culture Medium

Ion concentrations (Si, P, and Ca or Mg) were measured for composite scaffolds and bulk MBGs (0.02 g) during incubation in 2 ml cell culture medium containing 10% fetal calf serum (FCS) and 100 U·ml^−1^ penicillin and 100 μg ml^−1^ streptomycin (PS) over 14 days. The medium was collected and changed twice a week. All collected media were stored at 4°C until the analysis. The samples were diluted with 2% nitric acid (1:5) prior to the experiment. Ion concentrations were measured using inductively coupled plasma-optical emission spectroscopy (ICP-OES, Plasma Quant PQ 9000 Elite, Analytik Jena, Jena, Germany).

### 2.4 Effect of MBG Addition on Experiments Characterizing Embedded Cells

Human preosteoblasts (hOBs) were isolated from the femoral head of an osteoarthritic patient after total hip replacement at the University Hospital *Carl Gustav Carus* Dresden (Germany) after informed consent (approval by the Ethics Commission of TU Dresden, EK 303082014) as previously published (Bernhardt et al., 2020). The cells were expanded in α-MEM containing 15% fetal calf serum (FCS; Corning) and PS until the fourth passage and used for experiments.

#### 2.4.1 Effect of MBG on Observation of Metabolically Active Cells in Bioprinted Scaffolds

For identification of viable cells, hOBs were pre-labeled with Vybrant DiI staining solution (Thermo Fisher Scientific, Eugene, OR, United States) prior to mixing into inks. Cell suspensions containing 2.5 million of pre-labeled cells were added per 1 g of ink and mixed in manually with a spatula. Scaffolds were bioprinted in the same manner as in 2.2.2, and after crosslinking with CaCl_2_, α-MEM with 10% FCS and PS was added. The scaffolds were incubated at 37°C (5% CO_2_) for 21 days. The cell culture medium was changed twice a week. After 3, 7, and 14 days, the cell culture medium was removed, scaffolds were washed with Hank’s balanced salt solution (HBSS), and 0.6 µl of calcein-AM (Thermo Fisher Scientific) in 1 ml of cell culture medium was added. All samples were imaged using Keyence BZ-X810 fluorescence microscope.

#### 2.4.2 Effect of MBG Release Products on Cell Viability and Differentiation

Cell-free scaffolds printed as described in Section 2.2.2 were incubated in 2 ml α-MEM containing 10% FCS and PS. The conditioned medium was collected twice a week and osteogenic supplements (10^−7^ M dexamethasone, 10 mM β-glycerophosphate and 0.05 mM ascorbic acid 2-phosphate, all from Sigma-Aldrich) were added prior to putting it in contact with hOBs seeded in well plates 24 h before. After 3, 7 and 14 days, the cell layers were washed twice with PBS and stored at −80°C until performing the biochemical analysis with all the samples at the same time. For this, the cells were thawed at room temperature for 10 min and incubated with lysis buffer consisting of 1% Triton X-100 in PBS for 3 h on ice. The lysates were used for quantification of DNA and measuring of LDH and ALP activity. DNA quantification was performed using the Quantifluor dsDNA Kit (Promega, Madison, WI, United States) according to the manufacturer’s protocol. Fluorescence was read with a multifunction microplate reader (Infinite 200 PRO, Tecan, Switzerland) at excitation/emission wavelengths of 485/535 nm. The cell number was calculated using a calibration line constructed from defined cell numbers, which were frozen and lysed in the same way. The activity of intracellular lactate dehydrogenase (LDH) was quantified using the CytoTox 96 Non-Radioactive Cytotoxicity Assay (Promega, Madison, WI, United States): the lysates were diluted with the lysis buffer, the substrate solution was added, and absorbance was monitored for 5 min at 490 nm using the microplate reader. Slopes of absorbances were correlated with cell numbers using a calibration line. ALP activity was quantified by incubating each lysate with the substrate solution containing 1 mgml^−1^ 4-nitrophenylphosphate in 0.1 M diethanolamine, 0.1% Triton X-100, 1 mM MgCl_2_ (pH 9.8, all from Sigma-Aldrich) for 30 min at 37°C, and the enzymatic reaction was stopped with 1 M NaOH solution. Absorbance was measured at 405 nm using the microplate reader. Calibration line was constructed using different concentrations of 4-nitrophenol and finally, the amount of produced 4-nitrophenol within 30 min was related to the cell number.

#### 2.4.3 Effect of MBG on Cell Viability and Differentiation in Bioprinted Scaffolds

Bioprinted scaffolds containing hOBs were fabricated as described in Section 2.4.1, but without pre-labeling the cells, and were cultured at 37°C, 5% CO_2_ in the α-MEM supplemented with 10% FCS, PS, and osteogenic induction factors (10^−7^ M dexamethasone, 10 mM β-glycerophosphate, and 0.05 mM ascorbic acid 2-phosphate). After 24 h, 3, 7, and 14 days of culture scaffolds were washed twice with HBSS and stored at −80°C until performing the biochemical analysis with all the samples at the same time. The scaffolds were thawed at room temperature for 10 min and incubated with lysis buffer for 3 h; thereafter, the same procedure as described in Section 2.4.2 for cells seeded in well plates was followed.

#### 2.4.4 Effect of the MBG Structure and Release Products on Investigation of Cell Viability and Differentiation

**FIGURE 1:**
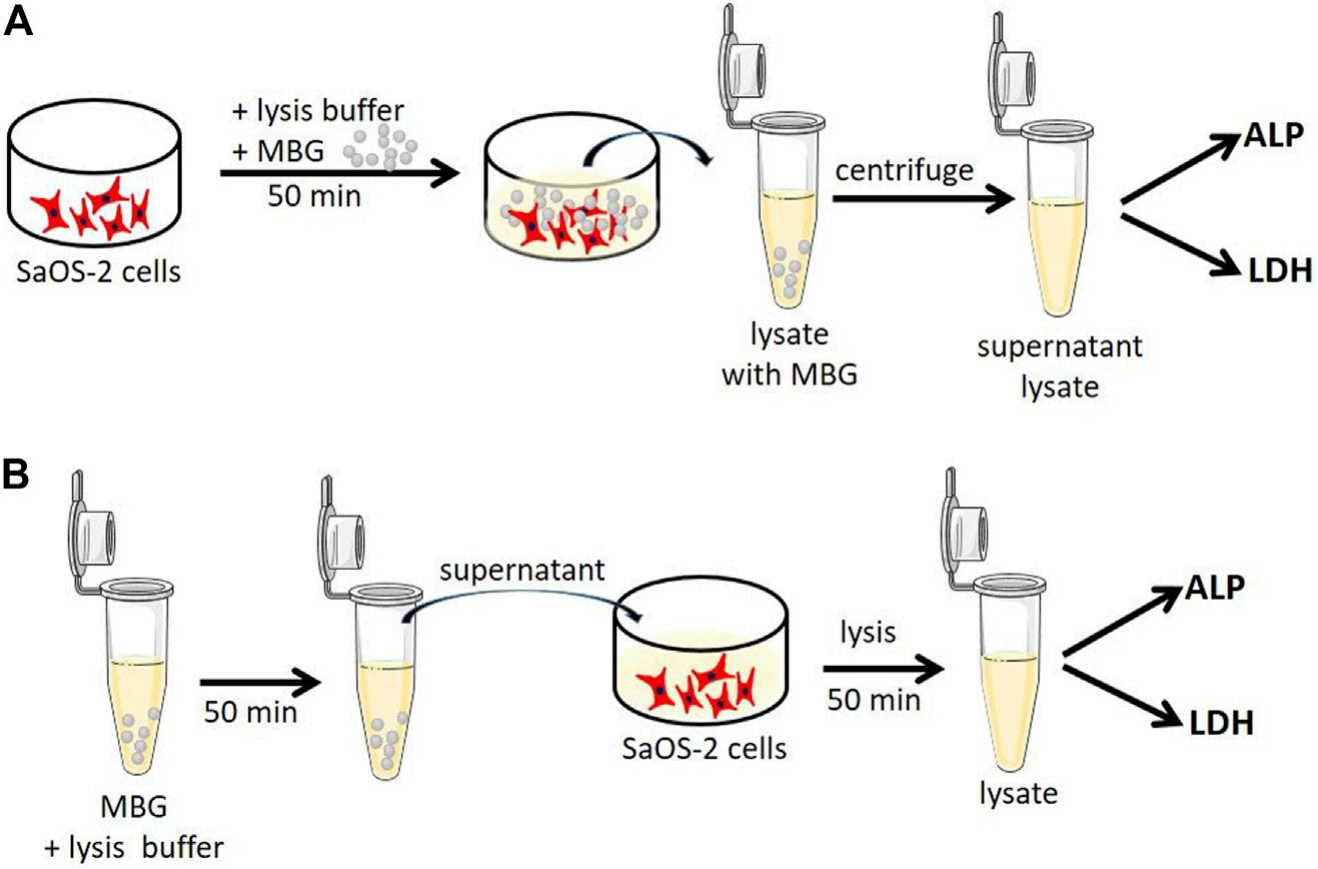
Schematic representation of experiments to investigate whether **(A)** cellular components (ALP and LDH) bind to MBG particles during lysis or **(B)** released components from MBG influence the ALP and LDH assay.

To investigate whether the presence of different MBGs had an effect on cell function or influenced the performed assays, additional experiments were conducted using the osteosarcoma cell line SaOS-2 (ACC 243, DSMZ, Braunschweig, Germany). These cells show high activity of both LDH and especially ALP after a short period of time and, therefore, they are a perfect control for the analysis. The cells were expanded in α-MEM supplemented with 15% FCS and PS. After one passage, the cells were seeded in TCPS, cultured in α-MEM supplemented with 10% FCS and PS at 37°C and 5% CO_2_ for 24 h, and frozen. At first, lysis was performed in the presence of MBG particles in order to evaluate whether potential binding of cellular components or release of certain ions influence the measurement (Figure 1A). At the time of the experiment, the cells were thawed at room temperature and placed on ice. 0.02 g (mass of MBG in one 4-layered scaffold) of CaMBG or MgMBG was placed in wells containing cells, and the lysis buffer was added. After 50 min of lysis, lysates containing MBG particles were collected in tubes and centrifuged to separate the lysates from the MBG particles. The supernatants were transferred to fresh tubes and used for measuring LDH and ALP activity as described above. In order to investigate whether the release products from MBG during lysis affected the analysis, the lysis buffer was at first incubated with 0.02 g of CaMBG or MgMBG for 50 min (Figure 1B). The samples were centrifuged and supernatants were transferred into fresh tubes and used for lysis of cells for 50 min, simulating ion release during the lysis procedure. LDH and ALP activity was measured as described above. For both experiments, control samples did not include MBG particles. Measured absorbances representing LDH or ALP activities were normalized to the control to show the variation in values when MBG was added.

### 2.5 Statistical Analysis

One-way analysis of variance (ANOVA) in GraphPad Prism 8 was performed to determine statistical significance of obtained data of shape fidelity and biochemistry studies.

## 3 Results

### 3.1 Modification of MBG With Mg Maintains the Channel Structure

**FIGURE 2:**
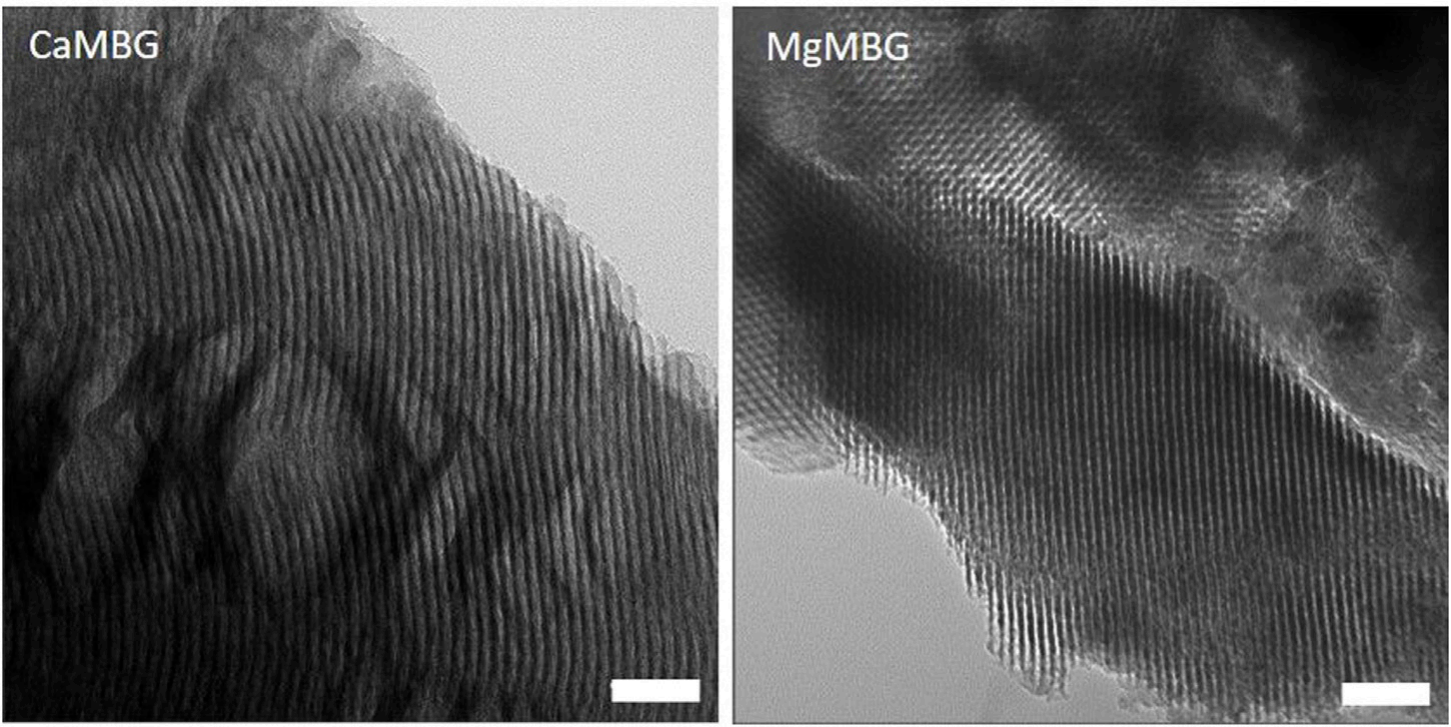
Mesoporous channel structure of MBG observed with TEM. Scale bars represent 50 nm.

Earlier analyses showed the channel structure of CaMBG (Guduric et al., 2021), which was confirmed in the present study. Exchange of the Ca^2+^ with Mg^2+^ in the glass network did not impair the channel structure. Representative TEM images of both CaMBG and MgMBG are shown in the Figure 2. Square and hexagonal structure of channels can be clearly seen on the presented image of MgMBG. The width of the channels of all glass particles was determined to be 5.59 ± 0.74 nm.

### 3.2 MBG Influences 3D Printing and Shape Fidelity of Composite Scaffolds Depending on Ion Modification

Square shaped scaffolds containing four layers were 3D-printed using different pressures and speeds adapted to the bioink composition (Table 1). MBG-free bioinks (3–6 algMC) had the lowest viscosity and, therefore, required the lowest pressure and the highest speed, while the composite containing CaMBG was completely opposite: higher viscosity requiring higher pressure and lower speed to achieve suitable shape fidelity of printed scaffolds. Viscosity and printing parameters of MgMBG were between the other two.

**FIGURE 3:**
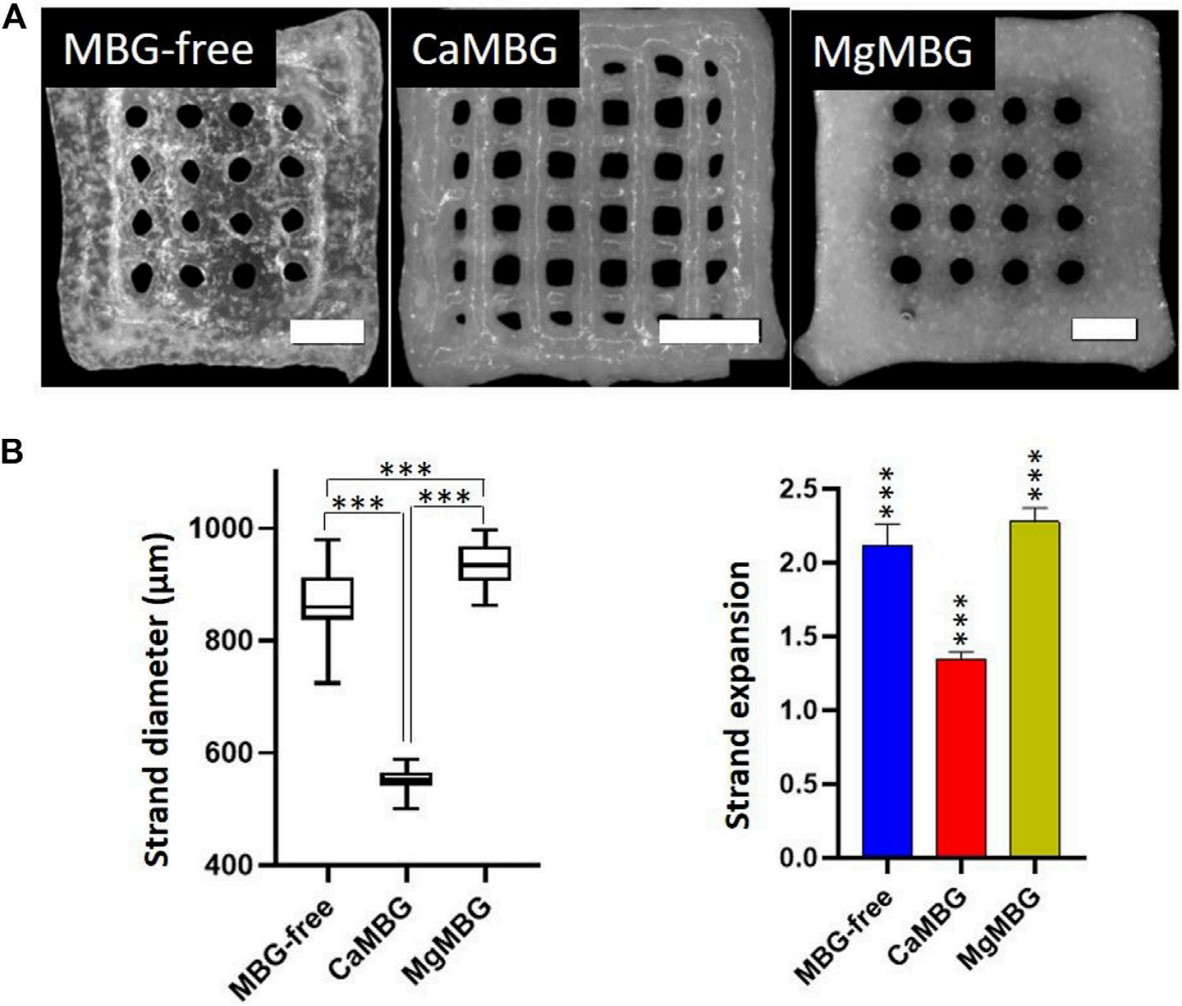
Printing properties of composite inks in comparison to MBG-free algMC (3–6)-ink. **(A)** 3D scaffolds printed using MBG-free and composites of 3–6 algMC with 7 wt% MBG (CaMBG or MgMBG). Scale bars represent 2 mm and **(B)** strand diameter of printed scaffolds and shape fidelity (strand expansion) in comparison with the ideal value (410 µm) with Tukey’s multiple comparison test. *n* = 24 (3 scaffolds with strands measured at 8 spots), *p* < 0.0001.

The low viscosity of 3–6 algMC bioink without MBG induced collapsing of strands during printing, forming round pores before finalizing the printing process. Similar effect was observed with MgMBG–algMC composite bioink, while CaMBG addition allowed the best stability of strands until the end of 3D printing. Representative images of produced scaffolds are shown in Figure 3A. Averaged strand diameters were measured on obtained images and strand expansions were calculated in relation to the theoretical value, representing the outlet diameter of the printing nozzle (410 µm) (Figure 3B). CaMBG–algMC composite inks could produce scaffolds with smaller strand diameters, the closest to the theoretical value (410 µm); the strand expansion was calculated to be 1.34 ± 0.04 (the theoretical value is represented by 1.0). MgMBG–algMC composite scaffolds could be printed with suitable shape fidelity represented by strand expansion of 2.28 ± 0.92. The size of strands of MBG-free algMC scaffolds was close to the MgMBG–algMC composite scaffolds with strand expansion of 2.12 ± 0.14. Significant differences in strand diameters were observed between all the prepared bioinks. MBG-free and MgMBG–algMC composite inks allowed the printing of scaffolds with approximately twice lower shape fidelity. All calculated shape fidelities were significantly different from those of the control.

As observed earlier, crosslinked CaMBG–algMC composite scaffolds were the least stable ones in cell culture conditions (Guduric et al., 2021). They started breaking apart after culture of couple of days in the cell culture medium or HEPES and could not completely last longer than 14 days, with very gentle manipulation. MBG-free and MgMBG-containing algMC scaffolds were completely stable during culture.

### 3.3 Ion Exchange of MBG-Containing Composites With the Cell Culture Medium

**FIGURE 4:**
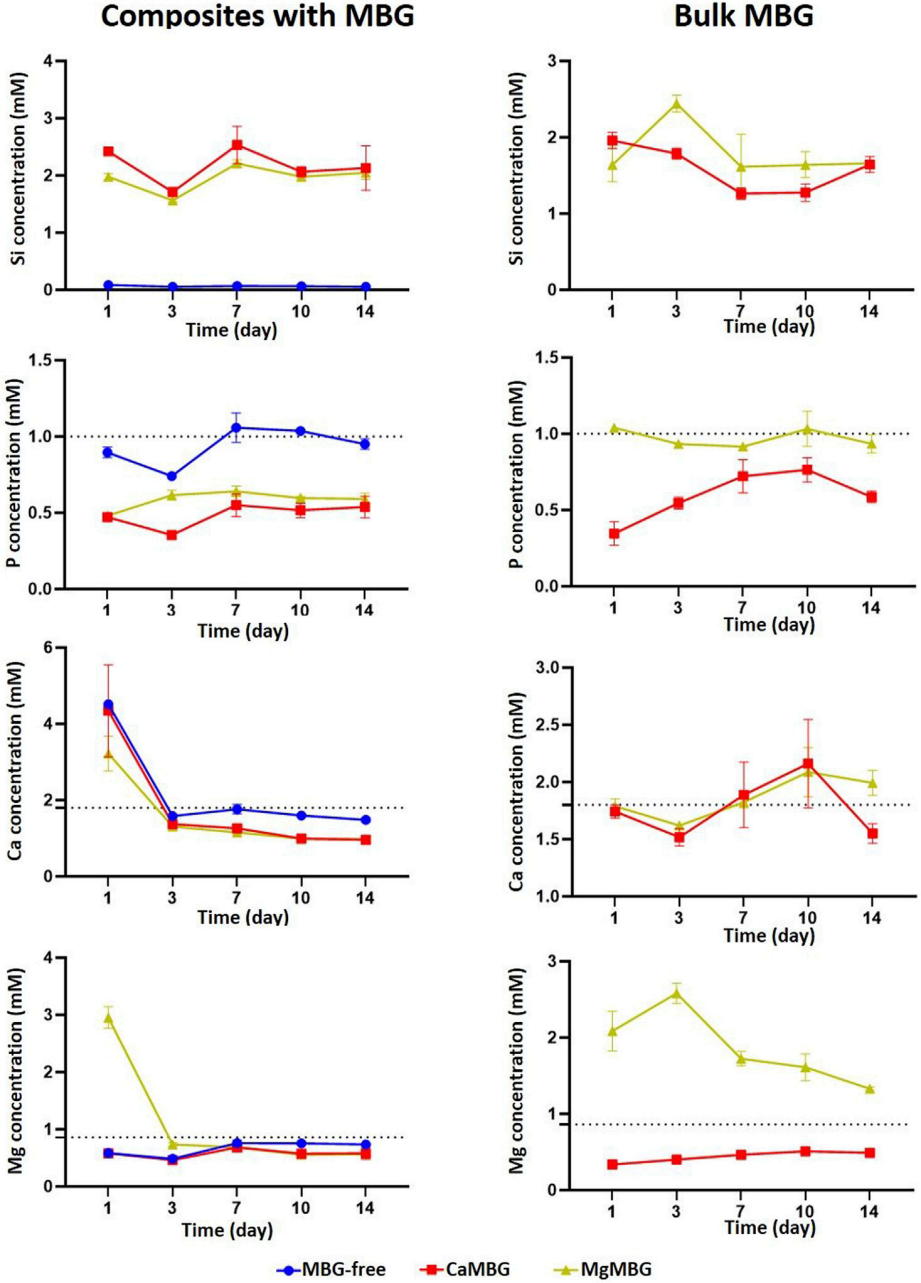
Ion release from algMC composites containing 7 wt % of MBG and from bulk MBG in the cell culture medium. Dotted lines represent concentrations of ions in cell culture medium. *n* = 3 scaffolds.

Concentrations in the cell culture medium of all ions present in MBG networks from composite scaffolds and bulk MBG powders over 14 days are presented in the Figure 4. Dotted lines represent concentrations of ions in the cell culture medium. The release profile of Si was similar for both composite scaffolds, with slightly lower concentrations in the case of MgMBG. As expected, released concentrations of this ion from bulk glasses were higher than from composites with higher release in the case of MgMBG. There was no detected release of phosphorus from composite scaffolds, but an uptake from the cell culture medium. The uptake was higher in the case of bulk glasses and for both types of samples, it was higher with CaMBG. High released concentration of Ca was measured at the first time point, but it probably corresponds to a remaining concentration of crosslinking solution. After that, an uptake was observed until the end of the experiment. This uptake was stronger in the case of bulk glasses, but the experiment ended with the low release if this ion from CaMBG samples. High concentration of magnesium was released from the MgMBG–algMC composite scaffolds only after 24 h and no release of this ion was detected at later time points. In the case of bulk MgMBG, the highest released concentration of magnesium was measured after 3 days and the release continued during 10 days. Bulk CaMBG induced constant uptake of this ion over 14 days.

### 3.4 Release Products of Composite Scaffolds Increase Viability and Differentiation of Human Preosteoblasts

**FIGURE 5:**
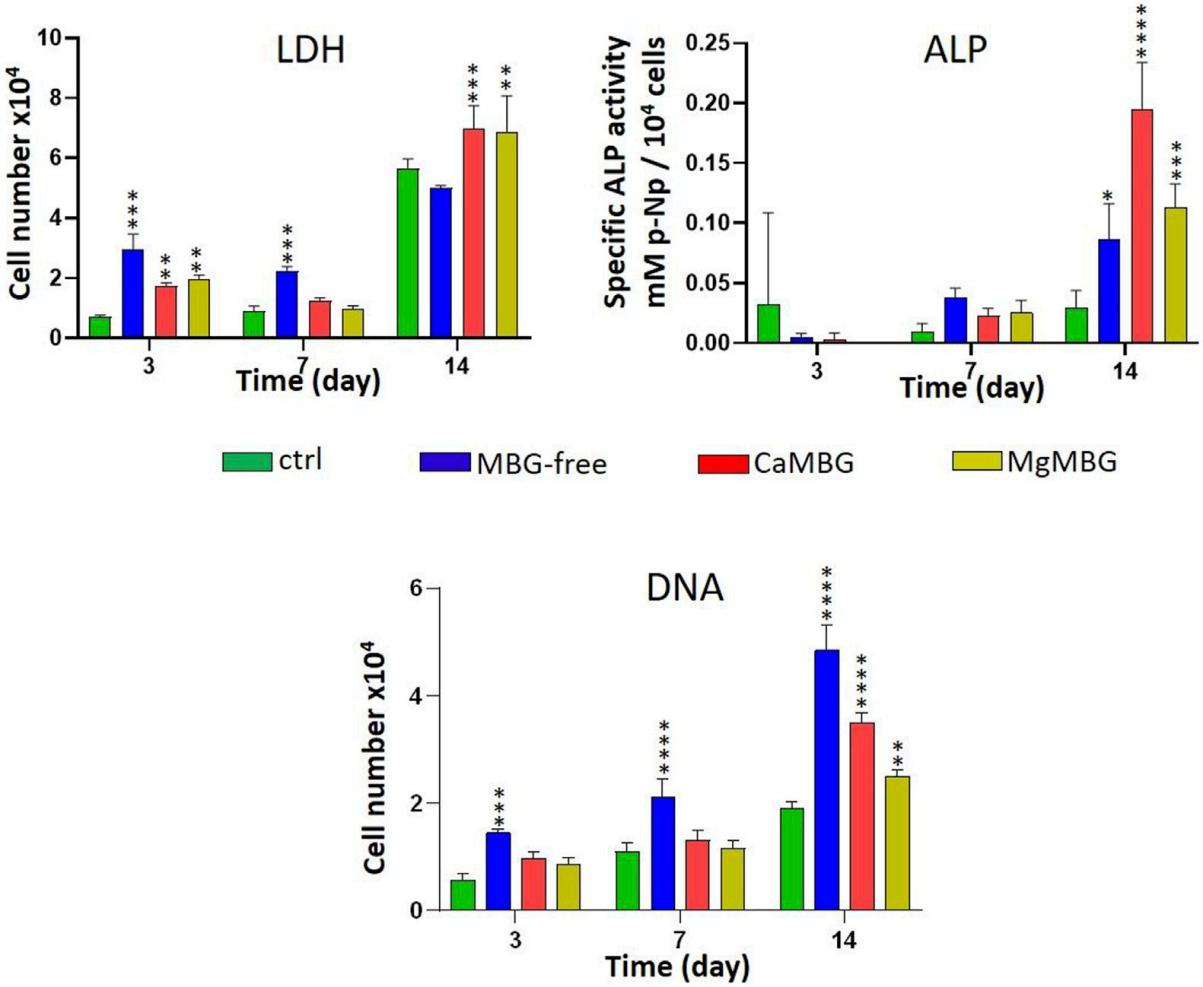
Effect of composite scaffold-release products on cell viability (LDH), differentiation (ALP) and DNA quantity of hOBs. Statistical analysis is performed in comparison to the control with Dunnett’s multiple comparison test. *n* = 3, *p < 0.05, **p < 0.005, ***p < 0.0005 and ****p < 0.0001.

Number of hOBs increased over time in the presence of the release medium of all the samples, as indicated by the LDH activity measurement shown in the Figure 5. At early time points, measured LDH activity in the presence of all extracts was increased in comparison to the control (cell culture medium). However, there was no positive effect observed in the presence of MBG compared to MBG-free samples. At later time points, no significant difference was observed between the control and MBG-free extracts, but the presence of MBG showed a positive effect. ALP activity, representing osteogenic differentiation, was increased over time and was significantly higher after 14 days, compared to that of the control. The highest value and significant difference was measured for CaMBG samples. Quantity of DNA showed lower cell number in the case of MBG-containing samples than MBG-free samples. After 14 days, measured DNA quantity was significantly higher in all the samples than in the control.

### 3.5 Pre-Labeling of Cells as a Solution to Study Cell Viability Using Fluorescence Imaging

**FIGURE 6:**
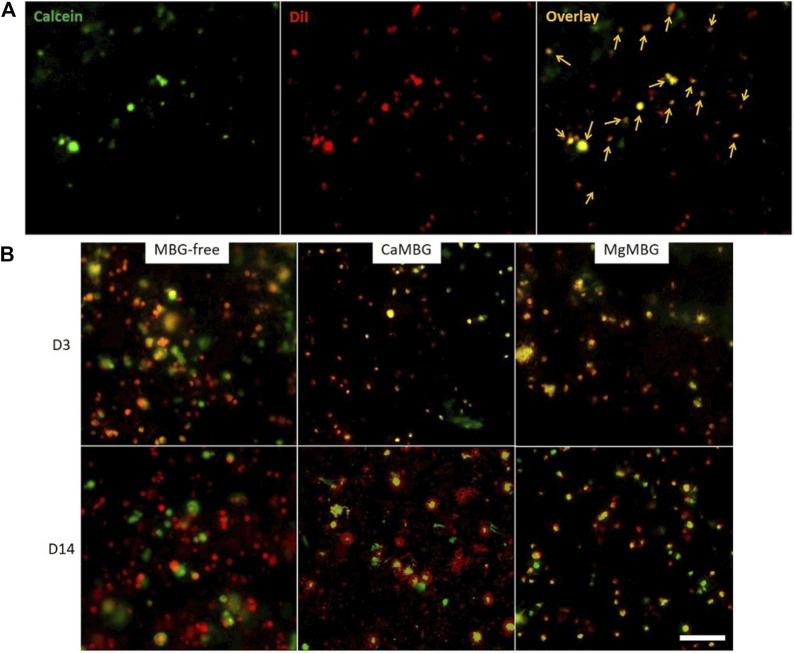
Visualization of metabolically active cells in MBG-containing bioprinted scaffolds. **(A)** Pre-labeled cells (with DiI in red) in bioprinted composite scaffolds stained with calcein (green). Orange arrows point on metabolically active cells showing signals in both red and green channels. **(B)** Pre-labeled cells in bioprinted scaffolds containing CaMBG or MgMBG stained with calcein during 14 days of cell culture (overlay of green and red channels). Scale bar represents 100 µm.

When the cells were pre-labeled with Vybrant DiI prior to 3D bioprinting (appearing in red in the Figure 6) and then stained with calcein-AM, both metabolically active cells and MBG particles expressed green signals. Red signals corresponding to MBG particles were visible only at very high exposure times together with the background. Therefore, it was possible to avoid their appearance and to visualize only the cells in the red channel. Objects on overlay images showing green and red signals at the same time correspond to metabolically active cells in bioprinted scaffolds. Solitary green signals come from MBG particles, while solitary red signals correspond to dead cells. MBG particles of particular shape (not specific for cells) appear in green channels (Figure 6A). Representative overlays of both green and red channels are shown in the Figure 6B for two different time points after staining composite and MBG-free bioprinted scaffolds. Orange color in overlay images arises from metabolically active cells (green and red signals together). In all tested samples, the cells were metabolically active during 14 days of culture.

### 3.6 Biochemical Assays Are Affected by MBG Due to Its Binding Capacity for Different Molecules and Its Release Products

**FIGURE 7:**
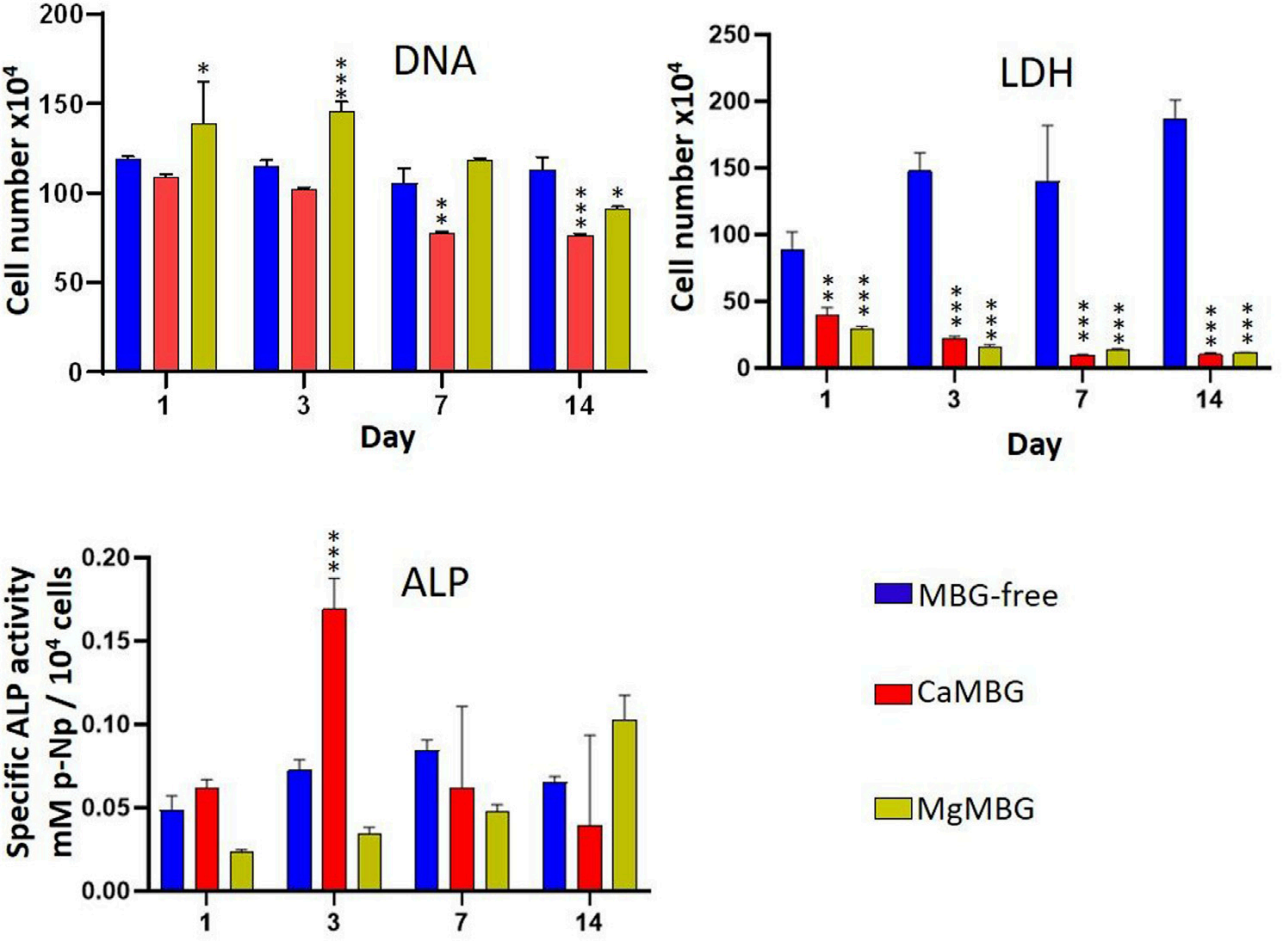
Cell viability (LDH) and differentiation (ALP) of hOBs in bioprinted composite scaffolds. Statistical analysis is performed in comparison to MBG-free samples at respective time points using Dunnett’s multiple comparison test. *n* = 3, *p < 0.005, **p < 0.0005 and ***p < 0.0001.

Bioprinted constructs of hOBs embedded in MBG-free algMC or MBG–algMC composites were subjected to different assays to quantitatively evaluate cell viability (LDH activity), cell number (DNA) and osteogenic differentiation (ALP activity), and obtained results are shown in the Figure 7. LDH activity of hOBs in MBG-containing constructs was significantly lower than that in the MBG-free constructs (without significant difference between composites). This result does not correspond to our microscopic observations of metabolically active cells as shown in Figure 6B. Quantification of cell number by measuring the DNA content after lysis revealed no significant differences between MBG-containing and MBG-free constructs and was in correspondence with our microscopic observations after staining with calcein-AM. Specific ALP activity, which was quantified as an early marker of osteogenic differentiation, was apparently affected by the presence of MBGs in the composites: highest ALP activity was detected for CaMBG composites with its maximum after 3 days of differentiation, while MgMBG containing composites revealed maximum ALP activity after 14 days.

**FIGURE 8:**
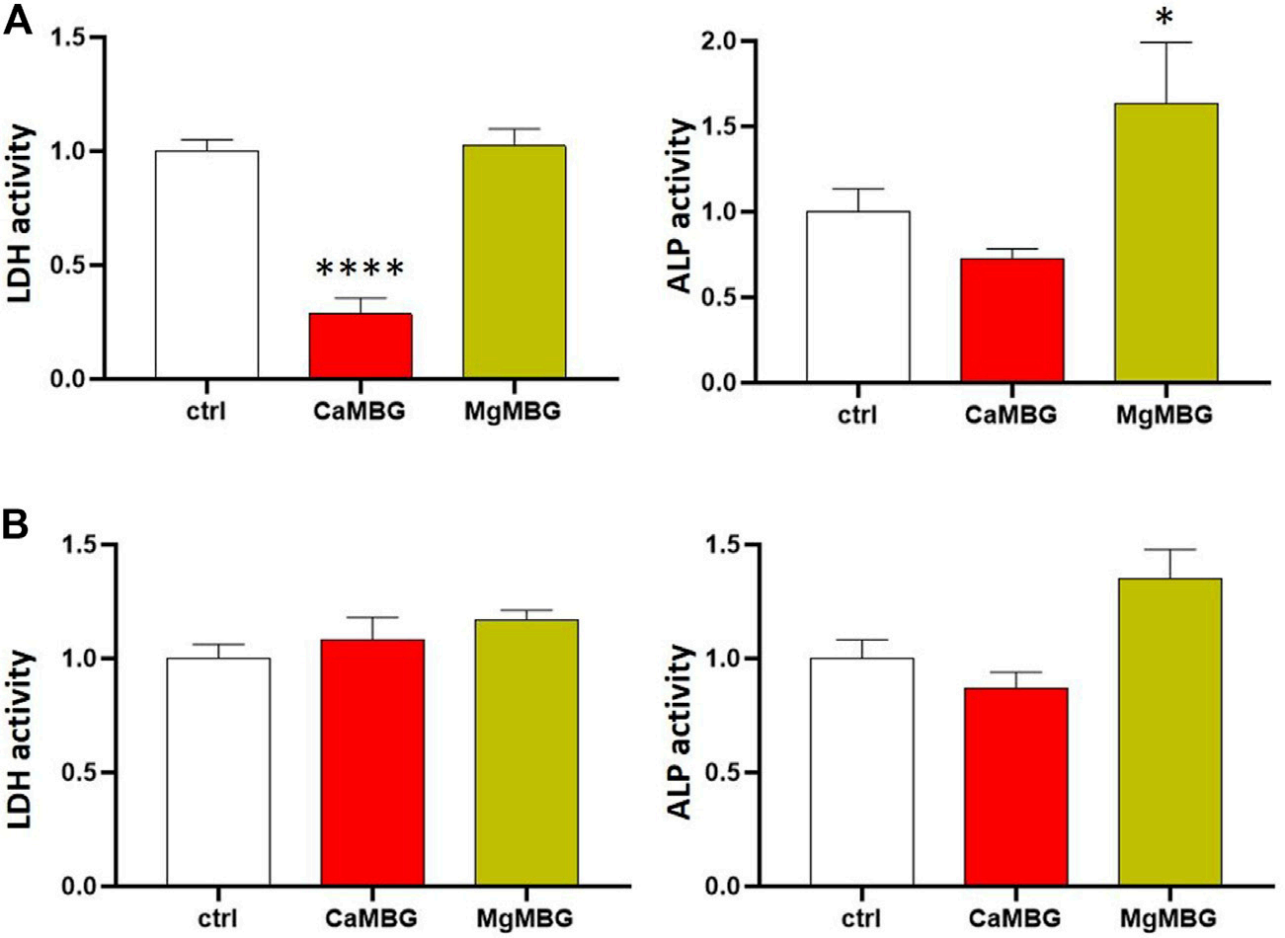
Effect of CaMBG and MgMBG on evaluation of cell viability (LDH activity) and differentiation (ALP activity) of SaOS-2 cells. Values are normalized to control (activity = 1), representing lysis buffer without MBG. Statistical analysis is performed in comparison with the control using Dunnett’s multiple comparison test with *n* = 3. **(A)** Effect of the MBG mesoporous structure (*p < 0.05 and ****p < 0.0001). **(B)** Effect of the release products of MBG.

To investigate a possible effect of MBGs on the performed assays, additional experiments were conducted (Figure 1). Two questions were to be answered: 1) Do ALP or LDH proteins bind to MBGs during lysis? and 2) do MBG dissolution products, which are released during lysis of the constructs, influence the assays? To answer the first question, osteoblasts cultivated in the monolayer were lysed in the presence and absence of MBG particles. LDH activity was significantly reduced after lysis in the presence of CaMBG, while with MgMBG, no significant changes were observed (Figure 8A). ALP activity was reduced in the presence of CaMBG and significantly increased in the presence of MgMBG. To address the second question, MBG particles (CaMBG and MgMBG) were incubated in lysis buffer which were subsequently used to lyse osteoblasts in monolayer culture. LDH and ALP assays were performed with those lysates compared to lysates which were generated with non-modified lysis buffer (Figure 8B). LDH activity was not significantly changed in the presence of MBG dissolution products. However, ALP activity was significantly increased in response to dissolution products of MgMBG, while the dissolution products of CaMBG did not show a significant effect (Figure 8B).

## 4 Discussion

Alginate-based hydrogels have been commonly used as bioinks. They can be easily modified by combination with different materials, changing not only final printing and mechanical properties but also cell responses in bioprinted scaffolds (Jungst et al., 2016). Alginate-based hydrogels can be easily combined with particulate materials, such as bioactive glasses, making them processable with 3D printing technologies. Bioactive glasses (BGs) have been combined with hydrogels affecting gelation, rheological, mechanical and degradation properties of final composites, depending on their initial composition and concentration (Zeimaran et al., 2021). Furthermore, the addition of BG affects shape fidelity, which represents the difference of the printed constructs to the related designed 3D model. We observed in this study that initial composition of the glass can induce the pre-crosslinking effect and improve shape fidelity as in the case of Ca-containing glass. Besides the effect of the BG and MBG on physicochemical properties of composite hydrogel based-inks, its addition to bioinks also affects the embedded cells. These effects have been reported with BG (Heid and Boccaccini, 2020; Zeimaran et al., 2021). Besides very recent studies (Guduric et al., 2021; Tavares et al., 2021), there is lack of data in literature about bioprinted cells within the MBG-hydrogel composites. One major reason for this is probably the difficulties to investigate cell behavior in the presence of MBG particles because of their interference with the assays. Hence, our aim here was to examine certain problems faced when performing different evaluation experiments when MBG is present in bioinks and to suggest possible solutions.

Two common ways to examine cell viability are simultaneous staining of live and dead cells with calcein-AM and ethidium homodimer-1, respectively (live/dead assay), and biochemical quantification of metabolic activity by measuring intracellular lactate dehydrogenase (LDH) activity (Jia et al., 2016, 3; Specian et al., 2016; Kumar et al., 2018; Bernal et al., 2019; Hauptstein et al., 2020; Schmidt et al., 2020). Previous studies dealing with glass-containing composite bioinks and observing metabolically active cells with live/dead assay used very low amounts of BG or MBG (0.1 and 0.5 wt%) (Leite et al., 2016; Tavares et al., 2021). However, they did not present a negative control to exclude possible signals of the glass. The same problem could arrive with other stainings, for example, HA-specific fluorescence staining, as applied in the study of Tavares et al. (Tavares et al., 2021). From our own experience, it is not possible to investigate cell viability in MBG-containing composite scaffolds by performing a commercial Live/Dead assay due to the appearance of signals of MBG particles in both green and red channels, resulting in difficulties to distinguish cells from MBG particles. This effect is especially strong with higher amounts of MBG, as used in the present study (7 wt %). It happens probably due to the mesoporous structure, specific for high binding capacity (Migneco et al., 2020), knowing that this effect was not observed when bioactive glass (not MBG) was included in a hydrogel (Leite et al., 2016). This is most likely the reason for the lack of studies presenting results based on fluorescence imaging of cells embedded in MBG-containing bioinks. In the present study, we suggest a solution for this problem by pre-labeling cells with Vybrant DiI staining solution prior to bioprinting and then staining only with calcein-AM. This resulted in metabolically active cells visible in both green and red channels while MBG particles were visible only in green. It is possible that other experiments based on fluorescence staining of cells in MBG-containing bioprinted scaffolds could be performed with pre-labeled cells. However, this should be verified with each single experiment. A negative control would always be necessary to avoid false interpretation. Still, the quantification is challenging, especially in cases of high cell densities.

On the other side, quantification of DNA and measuring of LDH activity provide quantitative cell viability results. At first, we performed the indirect assay with the release media and cells seeded in well plates in order to exclude the effect of MBG particles on the assays. Increased values for LDH were observed, without an important increase in DNA quantity. It suggested that release products (ions) really affected cell behavior.

In the direct measurement of DNA quantity and LDH and ALP activity of cells in bioprinted scaffolds, a surprising effect was observed. Measured absorbance for LDH activity in bioprinted MBG-containing scaffolds was very low, which was opposite from our observations of metabolically active cells stained with calcein/DiI in corresponding samples, suggesting that MBG did not negatively affect cell behavior but probably interfered with the assay itself. This assumption is further supported by the results obtained by DNA quantification as here the cell numbers in MBG-containing constructs were not significantly lower than those in MBG-free samples. To investigate this hypothesis, additional experiments were conducted in which MBG particles were only present during lysis but not during cultivation. SaOS-2 cells show high ALP activity (Farley et al., 1989) and can, therefore, be used as a positive control. After the experiment, it was not clear, especially in the case of LDH measurement, whether the MBG mesoporous channel structure, confirmed for both glasses with TEM, or products released during lysis were responsible for such results, especially in the case of CaMBG composites. To investigate that, the same amounts of MBG powders were at first incubated with the lysis buffer, which was after centrifugation (to remove MBG particles), transferred onto cells for lysis. In this case, release products from MBG were present in the lysis buffer. Measured LDH activities were comparable for all the samples and control, suggesting that the release products are not responsible, indicating binding of released LDH in the case of CaMBG. However, it stayed unclear why this effect was not observed in the presence of Mg instead of Ca in the MBG, suggesting that it is possible that not only the structure but also the composition of the glass affects the assay. In addition, it was obvious that release products of the tested MBG did not have any effect on LDH activity measurement.

Mg is a co-factor of ALP and, therefore, a necessary component of the substrate buffer. Increasing concentrations of this ion result in increasing values measured for ALP activity (Sorimachi, 1987; Olorunniji et al., 2007). Therefore, it was expected that Mg^2+^ ions released from MBG during lysis resulted finally in elevated ALP activity. When it was released from MBG in the lysis buffer, increased ALP activity for approximately 50% was measured. These findings show that the presence of MBG particles and certain modifying ions, released during cell lysis, strongly disturb established assays, producing false results as demonstrated herein by a seeming decrease or increase of LDH or ALP activity, respectively. Performing pre-tests with each MBG-containing composite type is necessary to avoid misinterpretations. In addition, it is possible to calculate factors for final data treatments in the cases where the effect is not very strong (e.g., ALP activity). However, usage of factors might be connected with the risk of systematic errors. For the LDH measurement, where the effect on the assay is very strong, DNA quantification could be an alternative option to calculate cell number. Finally, different measurement principles can be chosen to study metabolic activity, such as monitoring oxygen consumption of cells by nanosensors integrated in the bioink (Trampe et al., 2018).

The analysis of MBG degradation in composite bioinks can also lead to inconclusive results. Release of silicon, which is responsible for building of glass network, is actually the main indicator of glass degradation. Surprisingly, the difference between release profiles was observed between two different MBGs, even though the initial amounts of Si were the same. The reason for this could be in the possibility of Mg to act as a network builder as well (together with Si) and not only as a network modifier (Jones and Clare, 2012). In addition, crosslinking of oxygen in the glass network is stronger with Mg^2+^ than Ca^2+^(Jones and Clare, 2012), making it more stable, which could explain faster degradation of CaMBG in composite scaffolds, represented in higher released concentration of silicon. Low measured concentrations of certain ions do not always mean that they are not released at all. They might be released and then taken up by other compounds present in the release medium. Additionally, ions can also be taken up from the cell culture medium. This effect was observed with P, Ca, and Mg, which are actually present in the cell culture medium. The uptake of all these ions was stronger in the case of CaMBG for both composite scaffolds and bulk glasses. The reason for this could be in the precipitation of calcium phosphate–like structures, which is increased with increased concentration of Mg (N. Gunawidjaja et al., 2012; Tabia et al., 2019). This was not the case with bulk MBG, suggesting that the bioink itself has a strong effect on the release of therapeutic ions present in the MBG network. High concentration of Mg was measured for composite scaffolds only at the first time point and then it seemed as though the release completely stopped. From the measured concentrations for this ion in the case of bulk MgMBG, it was obvious that it was released over time. Hence, it is not possible to investigate degradation behavior of MBG within composite bioinks in the cell culture medium based only on measurement of concentrations of released ions due to strong effect on the bioink itself.

## 5 Conclusion

Even though modification of MBG with Mg did not disturb the mesoporous channel structure, it affected properties of composite bioinks. The hydrogel component of the ink strongly affected release of ions, leading to inconclusive results about MBG degradation. Furthermore, the presence of MBG in the bioink disturbed conduction of cell viability investigation using live/dead assays. Pre-labeling of cells prior to bioprinting allowed visualization of metabolically active ones expressing both red and green signals after staining with calcein-AM. The MBG structure, as well as certain release products, strongly affected assays of LDH and ALP activity, without affecting quantification of DNA, suggesting calculation of signal increase/decrease factors for each MBG composition or using alternative methods to study the metabolic activity of cells. Taken together, our findings point out possible misleading results which can appear during assays on bioprinted composite scaffolds containing MBG with suggestions which can help interpreting the data in future experiments.

## Data Availability

The raw data supporting the conclusions of this article will be made available by the authors, without undue reservation.
